# Investigation of smoking cessation intention and the associated factors in patients diagnosed with chronic obstructive pulmonary disease: A cross-sectional study

**DOI:** 10.18332/tid/214790

**Published:** 2026-02-13

**Authors:** Sibel Dogru, Hatice T. Akbayram, Sema Aytaç, Özlem Ovayolu

**Affiliations:** 1 Department of Pulmonary Diseases, Faculty of Medicine, Gaziantep University, Gaziantep, Türkiye; 2 Department of Family Medicine, Gaziantep University, Gaziantep, Türkiye; 3 Department of Nursing, Faculty of Health Sciences, Gaziantep University, Gaziantep, Türkiye

**Keywords:** intention to quit smoking, chronic obstructive pulmonary disease, COPD Assessment Test, Fagerström test for nicotine dependence

## Abstract

**INTRODUCTION:**

Intention to quit is an important early step and a strong predictor of smoking cessation success. In this study, individuals with chronic obstructive pulmonary disease (COPD) were evaluated with a newly developed scale for patients' intention to quit smoking using the current COPD staging system.

**METHODS:**

This cross-sectional study was conducted in Gaziantep, Turkey, from 1 March to 1 December 2024, and included 160 people with COPD who currently smoke, to investigate factors associated with smoking cessation intention using a questionnaire. Patients attending the chest diseases outpatient clinic were evaluated through face-to-face interviews using a structured questionnaire assessing demographic, clinical, and disease-specific measures. Shortness of breath was assessed using the modified Medical Research Council (mMRC) score, and COPD symptoms were evaluated using the COPD Assessment Test (CAT). COPD staging was performed according to the Global Initiative for Obstructive Lung Disease (GOLD) 2023 guidelines. Additionally, the Fagerström test for nicotine dependence and the Smoking Cessation Intention Scale were employed. GOLD staging (A, B, E) was defined as: GOLD A (≤1 non-hospitalized exacerbation, mMRC 0–1, CAT <10); GOLD B (mMRC ≥2 and/or CAT ≥10); and GOLD E (≥2 moderate or ≥1 hospitalized exacerbations).Descriptive statistics, non-parametric tests (Mann-Whitney U and Kruskal-Wallis), and Spearman’s rho correlation were used. In addition, generalized linear regression model (GLM) was applied.

**RESULTS:**

The mean age of the patients was 59.43 ± 10.54 years; 148 patients (92.5%) were male, and 104 patients (65%) had primary education. A weak but significant positive correlation was found between the Smoking Cessation Scale scores and both CAT (r=0.245, p=0.002) and mMRC scores (r=0.164, p=0.039). Lower cessation scores were significantly associated with single status (p=0.009), no quit attempts in the last year (p<0.001), lack of information about smoking cessation methods (p=0.016), absence of intention to quit smoking (p<0.001), lack of knowledge that smoking causes heart disease (p=0.035), and GOLD stage A (p=0.001). In the multivariate GLM analysis conducted with eight variables that were found to be significantly associated with the smoking cessation intention score in univariate analyses, three factors remained statistically significant: being married compared to being single (B=4.958; 95% CI: 1.203–8.714, p=0.010), having knowledge about smoking cessation methods compared to not having such knowledge (B=2.432; 95% CI: 0.192–4.672, p=0.033), and having the intention to quit smoking compared to lacking such intention (B=3.327; 95% CI: 1.117–5.536, p=0.003).

**CONCLUSIONS:**

Physicians should assess quit-smoking intention factors in COPD patients who continue smoking and should consider these factors in cessation interventions and referrals.

## INTRODUCTION

Smoking and tobacco use are the leading causes of preventable desease^1^. The World Health Organization (WHO) reports that an estimated 1.25 billion people globally are current users of tobacco products, with tobacco-related diseases accounting for over 8.7 million deaths annually^1^. Reducing these risks for smokers is an important public health goal. Smoking constitutes a modifiable environmental risk factor for chronic obstructive pulmonary disease (COPD), a respiratory disease characterized by progressive respiratory symptoms, permanent airflow restriction, and exacerbations^2^. It has been found that the incidence rate of COPD increased by 43.4% from 1990 to 2019^3^. Despite this, 38% of patients with COPD continue to smoke^4^.

Intention to quit smoking is one of the basic steps in the smoking cessation process and is an important predictor of smoking cessation^5^. The intention to quit smoking is closely related to the success of smoking cessation^6,7^. In order for anti-smoking programs to be successful, it is important to consider the factors that influence the intention to quit smoking^8^. Studies have reported that age, socioeconomic level, education level, addiction level, previous cessation attempts, respiratory symptoms, and being aware of the health hazards of smoking are factors associated with the intention to quit smoking^9-11^. In addition, the literature states that understanding the factors that affect the intention to quit smoking among smokers may help encourage cessation^12^. In theories of health behavior, smoking cessation intention has been reported as the main prerequisite for smoking cessation attempts^13,14^. Smoking cessation intention has been assessed using various methodologies in general populations. Droomers et al.^15^ utilized a Likert-type scale grounded in the Theory of Planned Behavior. Both Myung et al.^16^ and Reddy et al.^9^ applied multi-category measures based on the Global Adult Tobacco Survey (GATS). Additionally, Islam et al.^12^ used a multi-item Likert scale to more comprehensively capture varying levels of smoking cessation intention. In COPD patients, however, smoking cessation intention was assessed using a dichotomous response format that inadequately captures the multidimensional motivational readiness characteristic of this population, as evidenced by the work of Melzer et al.^10^ and Fan et al.^17^, who emphasized the critical necessity of comprehensive behavioral assessment beyond binary measurement approaches. A recent study among patients with COPD employed the Motivation to Stop Scale (MTSS), a validated single-item measure developed in 2013 to assess varying levels of quit intention^18^. These findings highlight a significant methodological gap necessitating novel and validated measurement tool to investigate COPD-specific factors influencing smoking cessation intention.

Whereas COPD is evaluated in four stages in the Global Initiative for Obstructive Lung Disease (GOLD) guideline according to the intensity, severity and number of exacerbations of symptoms, it is divided into three stages as GOLD A, B and E in the 2023 GOLD guideline^19^. While several studies have classified the severity of COPD based on forced expiratory volume in one second (FEV_1_), there appears to be a lack of research examining smoking cessation intention according to the updated GOLD classification, particularly in relation to stages A, B, and E^10,17,18^. For this reason, it may be important for patient management to examine whether the intention of patients to quit smoking changes as the COPD category progresses.

A comprehensive understanding of the correlates of smoking intention among COPD patients who continue to smoke is essential for informing the design of targeted tobacco control interventions. Such insights can also guide public health policy development and support early prevention strategies in this high-risk population. There remains a need for studies employing newly developed and validated instruments that take into account the updated COPD staging system. Therefore, this study aimed to examine the relationship between smoking cessation intention and demographic characteristics, clinical features, and GOLD staging in COPD patients.

## METHODS

### Study design

This cross-sectional study was conducted in Gaziantep, Turkey from 1 March to 1 December 2024, and included 160 people with COPD who currently smoke, to investigate factors associated with smoking cessation intention using a questionnaire. Patients attending the chest diseases outpatient clinic were evaluated through face-to-face interviews. The study was reported in accordance with the STROBE checklist.

A questionnaire was created by the researchers by reviewing the literature^2,20-22^ and each patient was interviewed for about 10–15 minutes.

The population of the study consisted of patients with COPD who were admitted to the chest diseases outpatient clinic of a tertiary healthcare institution. In this study, the minimum sample size was calculated as 111 in line with the effect size of 0.5, α=0.05, 1-β=0.95 and 160 patients were included in the study.

### Inclusion and exclusion criteria

Patients diagnosed with COPD and who currently smoke, admitted to the chest diseases outpatient clinic, were included in the study. Those who had smoked at least 100 cigarettes in their lifetime and had smoked at least one cigarette in the last 30 days were defined as ‘active smokers’^21^. Patients with COPD who fell outside these criteria were not included in the study.

### Data collection tools

*Questionnaire*

In the first part of the questionnaire (25 questions), there are questions about the sociodemographic and clinical characteristics of the patients^2,20-22^. In the first part, the smoking history was calculated as pack-years (number of packs smoked per day × years). In the second part of the form, patients’ shortness of breath was evaluated using the modified Medical Research Council score (mMRC), and COPD symptoms were assessed using the COPD assessment test (CAT)^2^. The COPD phase was performed according to the Global Initiative for Obstructive Lung Disease (GOLD) 2023 guideline^19^. The third part included the Smoking Cessation Intention Scale^22^ and the Fagerström test for nicotine dependence (FTND), the validity and reliability of which had already been tested in previous studies in Turkish^23,24^.

*Smoking Cessation Intention Scale*

This scale, developed by Söyler et al.^22^, is a five-point Likert scale consisting of a single dimension and eight items; the minimum possible score on the scale is eight and the maximum score is 40. Rising scores indicate a greater intention to quit.

*Fagerström test for nicotine dependence (FTND)*

The scale was developed to determine the physical level of nicotine dependence^23^. The FTND consists of six questions. Answer choices are scored on a scale from 0 to 3. The points are added together to get a total score of 0–10. In this study, as a result of the scores obtained from the scale, those who scored <5 were evaluated as ‘mildly dependent’, those who scored 5–6 as ‘moderately dependent’, and those who scored ≥7 as ‘highly dependent’^25^.

*COPD staging*

Patients with 0 or 1 exacerbations not requiring hospitalization in the last year, mMRC scores of 0–1 and CAT scores of <10 were defined as GOLD stage A, and patients with mMRC scores of ≥2 and CAT scores of ≥10 were defined as GOLD stage B. Patients with ≥2 moderate exacerbations or ≥1 exacerbations requiring hospitalization in the last year were defined as GOLD stage E^19^.

*Ethics*

Permission for the study was obtained from the Gaziantep University Clinical Research Ethics Committee (Decision no: 63/2024, Date: 28 February 2024). All respondents, provided signed informed consent before participation. The study adhered to the Helsinki Declaration for research on human participants.

### Statistical analysis

The data were evaluated using the Statistical Package for the Social Sciences Ver. 25.0 (SPSS) software package. Descriptive statistics are presented as frequencies and percentages, and means and standard deviations. The Shapiro-Wilk test was used to check the conformity of continuous variables to normal distribution, and the Mann-Whitney U and Kruskal-Wallis tests were used to examine the difference between categorical variables. Correlations between continuous variables with non-normal distributions were examined using Spearman’s correlation coefficient (rho). As the dependent variable (Intention to Quit Smoking Scale) did not exhibit a normal distribution – even after transformation attempts – a generalized linear model (GLM) was used for multivariable analysis. Independent variables were selected based on their statistically significant associations with smoking cessation intention in univariate analyses. These independent variables were marital status, GOLD stage of COPD, intention to quit smoking (yes, no), smoking cessation attempt in the last year, getting information about smoking cessation methods, ‘can smoking cause heart disease’ (yes,no), CAT score, and mMRC score.

In all analyses were two-sided, and p<0.05 was considered statistically significant. The Cronbach’s alpha coefficient of the Smoking Cessation Intention Scale is 0.94325. In this study, Cronbach’s alpha coefficient of this scale was 0.874.

## RESULTS

The mean age of the patients was 59.43 (SD=10.54) years, 148 patients (92.5%) were male, and 104 patients (65%) were primary school graduates. The rate of tobacco product use other than cigarettes was low in 8 patients (5%), while the median age of smoking initiation was 17.55 (IQR: 15–20) years. The median number of cigarettes consumed per day was 20 (IQR: 12.5–25), and median pack-years were 40 (IQR: 20–52.5). The median number of smoking cessation attempts in the last 12 months was 1 (IQR: 1–2). Among the patients, 64 (40.0%) were classified as mildly dependent on nicotine (FTND <5), 42 (26.3%) as moderately dependent (FTND = 5–6), and 54 (33.8%) as highly dependent (FTND ≥7). Among the patients with COPD, 69 (43.1%) had GOLD A, 63 (39.4%) had GOLD B, and 28 (17.5%) had GOLD E. For the Smoking Cessation Intention Scale the median (IQR) score was 29 (IQR: 25–32), for the FTND 6 (IQR: 3–7), for the CAT 18 (IQR: 10–26), and for the mMRC 1.50 (IQR: 1–2) (Table 1).

**Table 1:** Sociodemographic, smoking-related, and clinical characteristics of study participants in a cross-sectional study of COPD patients attending the chest diseases outpatient clinic in Gaziantep, Türkiye, 2024 (N=160)

*Characteristics*	*Categories*	*n*	*%*
**Age** (years), mean (SD)		59.43	10.54
**Sex**	Female	12	7.5
	Male	148	92.5
**Income level**	Less than the minimum wage	108	67.5
	Minimum wage and above	52	32.5
**Education level**	University and higher	10	6.3
	High school	33	20.6
	Primary	104	65.0
	No formal education	13	8.1
**Age of smoking onset**, median (IQR)		17	15.0–20.0
**Total years smoked**, median (IQR)		42	31.5–48.5
**Cigarettes per day**, median (IQR)		20	12.5–25.0
**Pack-years**, median (IQR)		40	20.0–52.5
**Use of tobacco products other than cigarettes**	No	152	95.0
	Hookah	2	1.3
	Other	6	3.8
**Comorbidities**	Hypertension	25	48.1
	Coronary artery disease	8	15.4
	Lung cancer	1	1.9
	Diabetes mellitus	18	34.6
	Diagnosis of psychiatric illness	6	3.8
**GOLD stage of COPD**	A	69	43.1
	B	63	39.4
	E	28	17.5
**Emergency admission due to COPD in the last year**	Yes	76	47.5
	No	84	52.5
**Number of emergency admissions due to COPD in the last year**, median (IQR)		4	2–6
**Hospitalization due to COPD in the last year**	Yes	40	25.0
	No	120	75.0
**Number of hospitalizations for COPD in the last year**, median (IQR)		1	1–2
**An attempt to quit smoking**	Yes	105	65.6
	No	55	34.4
**Number of smoking cessation attempts in the last year**, median (IQR)		1	1–2
**Getting support to quit smoking**	Yes	74	46.3
	No	86	53.8
**Intention to quit smoking**	Yes	99	61.9
	No	61	38.1
**Knowledge about smoking cessation methods**	Yes	44	27.5
	No	116	72.5
**Sources of information about smoking cessation methods**	Written material	0	0.0
	Social media	3	6.8
	Television	6	13.6
	Other	12	27.3
	Health professional	23	52.3
**An attempt to quit smoking in the last year**	Yes	65	40.6
	No	95	59.4
**Knowing that smoking can cause a stroke**	Yes	115	71.9
	No	8	5.0
	I don’t know	37	23.1
**Knowing that smoking can cause heart disease**	Yes	132	82.5
	No	4	2.5
	I don’t know	24	15.0
**Knowing that smoking can cause lung cancer**	Yes	141	88.1
	No	2	1.3
	I don’t know	17	10.6
**The reason for continuing smoking**	Does not want to quit	32	20.0
	Wants to quit, but cannot	128	80.0
**Fagerström test for nicotine dependence**	Slightly dependent	64	40.0
	Moderately dependent	42	26.3
	Highly dependent	54	33.8
**Fagerström test for nicotine dependence score**, median (IQR)		6	3–7
**CAT score**, median (IQR)		18	10–26
**mMRC score**, median (IQR)		1.50	1–2
**Smoking Cessation Intention Scale score**, median (IQR)		29	25–32

CAT: chronic obstructive pulmonary disease assessment test. mMRC: modified Medical Research Council score. COPD: chronic obstructive pulmonary disease. IQR: interquartile range. Statistical significance at p<0.05.

**Figure 1:**
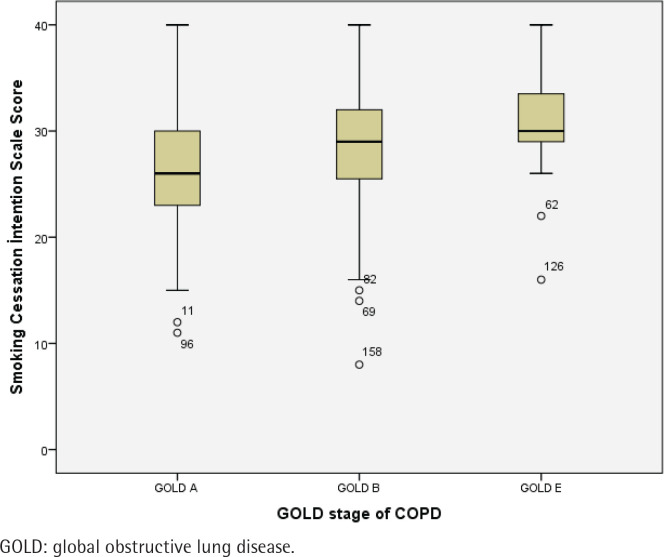
Intention to quit smoking scores in COPD patients according to the GOLD stage classification (N=160)

Single participants demonstrated significantly lower median Smoking Cessation Intention Scale scores compared to married participants, according to the results of the Mann–Whitney U test [median (IQR): 21 (17–32) vs 29 (25–32), respectively, p=0.009]. Patients with COPD GOLD stage A exhibited significantly lower median scores than those with GOLD stage B and E as shown by the Kruskal-Wallis test [26 (23–30) vs 29 (25–32) and 30 (29–34), respectively, p<0.05]. Participants without intention to quit smoking showed significantly lower median scores than those with quit intention as shown by the Mann–Whitney U test [25 (20–29) vs 30 (27–33), p<0.01]. Participants who had not attempted smoking cessation in the past year demonstrated significantly lower median scores than those who had made quit attempts as assessed using the Mann–Whitney U test [27 (8) vs 30 (5), respectively, p<0.01]. The score was significantly lower among those who did not receive information about smoking cessation methods compared to those who did, according to the results of the Mann-Whitney U test [28 (24–32) vs 29.5 (27–34), respectively, p=0.016]. Additionally, participants who were unaware that smoking causes heart disease had significantly lower median Smoking Cessation Intention Scale scores compared to those who responded ‘no’ or ‘yes’ , as shown by the Kruskal-Wallis test [25 (22–29) vs 26 (23–30) and 29 (25–32), respectively, p=0.035] (Table 2). Intention to quit smoking scores in COPD patients according to the GOLD stage classification are shown in Figure 1.

**Table 2:** Comparison of the characteristics related to illness and smoking with the median score of the Smoking Cessation Intention Scale in a cross-sectional study of COPD patients attending the chest diseases outpatient clinic in Gaziantep, Türkiye, 2024 (N=160)

*Characteristics*	*Categories*	*Smoking Cessation Intention Scale score Median (IQR)*	p*
**Sex**	Female	28 (20–31)	0.438^†^
	Male	29 (25–32)	
**Marital status**	Married	29 (25–32)	0.009^*†^
	Single	21 (17–32)	
**Income level**	Less than the minimum wage	29 (25–32)	0.634^†^
	Minimum wage and above	28 (20–32)	
**Education level**	University and higher	24 (17–28)	0.190^‡^
	High school	29 (25–32)	
	Primary	29 (25–32)	
	No formal education	28 (21–30)	
**Use of tobacco products other than cigarettes**	No	29 (25–32)	0.467^‡^
	Hookah	30.5 (29–32)	
	Other	28.50 (28–30)	
**Presence of comorbidities**	Hypertension	28 (25–30)	0.114^‡^
	Diabetes mellitus	29 (25–32)	
	Coronary artery disease	24.5 (22–26)	
**GOLD stage of COPD**	A	26 (23–30)	0.001^*‡^
	B	29 (25–32)	
	E	30 (29–34)	
**Emergency presentation in the last year**	Yes	29 (25–32)	0.107^†^
	No	28 (23–32)	
**Hospitalization in the last year**	Yes	29 (26–31)	0.845^†^
	No	28.5 (25–32)	
**Intention to quit smoking**	Yes	30 (27–33)	<0.001^*†^
	No	25 (20–29)	
**Smoking cessation attempt in the last year**	Yes	30 (28–33)	<0.001^*†^
	No	27 (22–30)	
**Getting support for smoking cessation**	Yes	29 (25–32)	0.362^†^
	No	29 (25–32)	
**Support for smoking cessation**	Nicotine patch	34 (30–37)	0.063^‡^
	Nicotine gum	30.5 (27–34	
	Varenicline	29 (36–30)	
	Psychological support	30 (27–35)	
	Other	28 (24–31)	
**Getting information about smoking cessation methods**	Yes	29.5 (27–34)	0.016^*†^
	No	28 (24–32)	
**Source of information about smoking cessation methods**	Face-to-face	32 (27–36)	0.233^‡^
	Television	27 (23–29)	
	Social media	29 (22–30)	
	Other	30 (29–32)	
**Does smoking cause paralysis**	Yes	29 (25–32)	0.073^‡^
	No	26 (23–31)	
	I don’t know	27 (7)	
**Can smoking cause heart disease**	Yes	29 (25–32)	0.035^*‡^
	No	26 (23–30)	
	I don’t know	25.50 (22–29)	
**Does smoking cause lung cancer?**	Yes	29 (25–32)	**0.050**
	No	28.50 (25–32)	
	I don’t know	24 (17–30)	
**Fagerström test for nicotine dependence**	Slightly dependent	29 (28–32)	0.059^*‡^
	Moderately dependent	28 (23–32)	
	Highly dependent	27 (22–32)	

† Mann-Whitney U test.

‡ Kruskal-Wallis test.

* Statistical significance at p<0.05. IQR: interquartile range.

Spearman correlation analysis between continuous variables and Smoking Cessation Intention Scale scores revealed weak but statistically significant positive correlations for CAT scores (ρ=0.245, p=0.002) and mMRC scores (ρ=0.164, p=0.039) (Table 3).

**Table 3:** Spearman correlation analysis between Smoking Cessation Intention Scale scores and continuous variables among COPD patients attending the chest diseases outpatient clinic in Gaziantep, Türkiye, 2024 (N=160)

*Variables*	*Smoking Cessation Intention Scale*	
	*rho*	p*
Age	0.017	0.828
Age of onset of smoking	0	0.998
Duration of smoking (years)	-0.011	0.893
Cigarettes per day	-0.119	0.135
Pack-years	-0.150	0.059
Number of emergency room admissions in the last year	0.057	0.623
Number of hospitalizations in the last year	0.088	0.589
Number of smoking cessation attempts in the last year	0.031	0.756
Fagerström test for nicotine dependence	-0.150	0.059
CAT score	0.245	**0.002**
mMRC score	0.164	**0.039**

Spearman correlation analysis was conducted due to the non-normal distribution of the data. rho: Spearman correlation coefficient. CAT: chronic obstructive pulmonary disease assessment test. mMRC: modified Medical Research Council score.

* p<0.05 was considered statistically significant.

A GLM was constructed incorporating eight variables significant in univariate analyses: marital status, GOLD stage of COPD, intention to quit smoking (yes, no), smoking cessation attempt in the past year, getting information about smoking cessation methods, ‘can smoking cause heart disease’ (yes/no), CAT score, and mMRC score (Table 4). The model results were statistically significant (p<0.001), explaining 14–26% of variance in intention scores (Cox and Snell R^2^=0.14; Nagelkerke R^2^=0.26). Three variables remained significant: married versus single status (B=4.958; 95% CI: 1.203–8.714, p=0.010), getting information about smoking cessation methods versus not getting information (B=2.432; 95% CI: 0.192–4.672, p=0.033), and having intention to quit smoking versus lacking such intention (B=3.327; 95% CI: 1.117–5.536, p=0.003).

**Table 4:** Adjusted multivariable generalized linear model results examining factors associated with Smoking Cessation Intention Scale scores in a cross-sectional sample of COPD patients attending the chest diseases outpatient clinic Gaziantep, Türkiye, 2024 (N=160)

*Variables*	*B*	*SE*	*Wald 95% CI*		*Hypothesis test*		
			*Lower*	*Upper*	*Wald χ^2^*	*df*	*p*
**Intercept**	20.464	4.0698	12.487	28.440	25.282	1	0.000
**Marital status**							
Married	4.958	1.9160	1.203	8.714	6.697	1	**0.010**
Single	0	.	.	.	.	.	.
**GOLD stage of COPD**							
A	-3.771	2.0746	-7.837	0.295	3.304	1	0.069
B	-2.808	1.7419	-6.222	0.607	2.598	1	0.107
E	0	.	.	.	.	.	.
**Getting information about smoking cessation methods**							
Yes	2.432	1.1428	0.192	4.672	4.528	1	**0.033**
No	0	.	.	.	.	.	.
**Can smoking cause heart disease**							
Yes	1.694	1.6798	-1.599	4.986	1.017	1	0.313
No	-2.127	3.0268	-8.060	3.805	0.494	1	0.482
I don’t know	0	.	.	.	.	.	.
**mMRC score**							
0	0.083	3.6283	-7.028	7.195	0.001	1	0.982
1	0.656	3.1953	-5.606	6.919	0.042	1	0.837
2	2.225	3.0155	-3.685	8.135	0.544	1	0.461
3	-0.187	2.9319	-5.934	5.559	0.004	1	0.949
4	0	.	.	.	.	.	.
**Intention to quit smoking**							
Yes	3.327	1.1271	1.117	5.536	8.711	1	**0.003**
No	0	.	.	.	.	.	.
**Number of smoking cessation attempts in the last year**	0.271	0.2729	-0.264	0.806	0.987	1	0.320
**CAT score**	0.023	0.0677	-0.109	0.156	0.120	1	0.729
**Smoking Cessation Intention Scale**	23.450	3.2364	17.892	30.734			

The multivariable model was adjusted for the following covariates: marital status, GOLD stage of COPD, intention to quit smoking, smoking cessation attempt in the last year, getting information about smoking cessation methods, ‘can smoking cause heart disease’, CAT score, mMRC score. Dependent variable: Smoking Cessation Intention Scale score. mMRC: modified Medical Research Council score. CAT: chronic obstructive pulmonary disease assessment test. Cox and Snell R²=0.14; Nagelkerke R²=0.26. SE: standard error.

*p<0.05 was considered statistically significant.

## DISCUSSION

This study revealed that the intention to quit smoking was lower among single patients, COPD GOLD stage A, low CAT and mMRC scores, lack of knowledge that smoking can cause heart disease, lack of knowledge of smoking cessation methods, absence of smoking cessation attempts in the last year, and no intention to quit smoking. In addition, multivariate analysis revealed that participants’ single marital status, lack of knowledge of smoking cessation methods, and no intention to quit smoking were associated with the smoking cessation intention score. These results clearly indicate that multiple factors related to smoking cessation intention should be considered, and a comprehensive approach should be adopted for patients with COPD.

Similar to previous studies, we found that sociodemographic characteristics such as age, sex, income level, and education level were not associated with intention to quit smoking^26,27^. However, one study reported that individuals with higher income level, younger age, and lower daily cigarette consumption were more likely to report a higher intention to quit smoking^16^. Differences in tobacco control programs between countries may modify the influence of sociodemographic factors such as age, sex, and income on smoking cessation intentions. In contexts with well-established tobacco control policies – such as education campaigns, taxation, and cessation support – sociodemographic factors may exert a more direct influence on quit intentions^26,27^. In contrast, in settings with weaker control measures, personal beliefs and social environments may play a more prominent role^16^.

We found that marital status was an independent factor associated with smoking cessation intention, and single individuals had less intention to quit smoking. However, previous studies have reported no significant association between marital status and cessation intention^15,16^. This difference can be explained by the fact that smoking cessation intention was evaluated in different ways in the previous studies. In one study, the intention to quit smoking was defined as the intention to quit within the next 3 months, whereas in another study, it was defined as the intention to quit within the next 6 months^15,16^. In the present study, the intention to quit smoking was evaluated cross-sectionally using the newly developed ‘Smoking Cessation Intention Scale’. In line with previous research, the association between marital status and smoking cessation intention suggests that strengthening social support – particularly for single or socially isolated individuals – may enhance motivation to quit^28^. Cessation programs should incorporate interventions targeting social connectedness.

In the present study, the intention to quit smoking was lower in patients who were GOLD stage A. Previous study has reported that patients in milder GOLD stages (GOLD I–II) were less likely to intend to quit smoking or achieve successful cessation^10^. However, these studies were conducted prior to the 2023 GOLD revision, which introduced the new Group E. To the best of our knowledge, no study to date has specifically examined smoking cessation intention in Group E, positioning our findings among the earliest contributions to this emerging area of research.

According to our results, the intention to quit smoking was weaker in patients who did not know that smoking caused heart disease. Contradictory results have been reported in the literature. Mohammadi et al.^29^ found that there was no significant relationship between smoking behavior and awareness of the harms of smoking. In an Australian study, it was reported that as the level of knowledge about the harms of smoking increased, the intention to quit smoking in the next 3 months it decreased^30^. In another study evaluating the intention to quit smoking, it was shown that perceiving the health hazards of smoking was significantly related to the intention to quit smoking^16^. For this reason, education to increase knowledge and awareness about the harms of tobacco in patients with COPD may be beneficial in increasing the intention to quit smoking in patients.

When the CAT and mMRC scores, which measure the intensity of symptoms, were low in patients with COPD, the intention to quit smoking was weak. Similar to our study, a recent study reported that patients with COPD with fewer respiratory symptoms had less intention to quit smoking^10^. This may be due to clinicians’ stronger approach to smoking cessation in patients with COPD with more intense symptoms. These results may suggest that patients with few symptoms should question their intention to quit smoking more carefully and that appropriate interventions are needed to increase the motivation to quit smoking.

In the current study, the intention to quit smoking was low in those who did not attempt to quit smoking in the last year. Different results have been reported in the literature on this subject. In a recent multicenter study, no association was found between smoking cessation intention and quit attempts^17^, whereas another study by Zhou et al.^30^ demonstrated a significant positive relationship. These varying findings suggest that the relationship between cessation intention and past quit behavior may differ across populations and study settings. Although our results point to a potential link, longitudinal studies are warranted to establish the direction and strength of this association. Nonetheless, enhancing smoking cessation support for patients with COPD may still help foster greater quit intention and may improve cessation outcomes over time.

We found that the level of nicotine dependence was high in most of the patients, but there was no significant difference between the level of dependence and the intention to quit smoking. Feng et al.^31^ and Danielson et al.^32^ reported that the desire to quit decreased as the level of nicotine dependence increased. However, in these studies, different criteria were used to evaluate the level of dependence and the intention to quit smoking. The use of different measurement instruments specifically, the use of the FTND and a validated multi-item intention scale in our study versus the Heaviness of Smoking Index and single-item or ordinal self-report measures in the studies by Feng et al.^31^ and Danielsson et al.^32^, may partially explain the inconsistencies in the observed findings. The findings of this study reveal that focusing solely on nicotine dependence levels may be insufficient in evaluating smoking cessation intentions. In clinical practice, this may suggest that, multidimensional approaches, independent of dependence severity, should be adopted when assessing patients’ intentions to quit smoking.

### Strengths and limitations

In this study, the fact that the intention to quit smoking was assessed using a new and validated scale, unlike previous studies, is one of the strengths of the study. We think this study will make significant contributions to the literature because it is the first to examine the relationship between the current intention to quit smoking scale and the new 2023 GOLD A, B and E staging.

The study has, however, several limitations. First, the cross-sectional design limits assessment of whether patients’ quit intentions resulted in actual cessation. Unmeasured confounders – such as psychological status, social support, and environmental factors – may also have influenced the results, warranting cautious interpretation. Second, because the number of female patients included in the study was small, the results between the sexes and intention to quit smoking were not statistically strong. Third, this single-center study conducted in Turkey limits the generalizability of findings to COPD patients in countries with different healthcare systems, sociocultural contexts, and tobacco control policies. Fourth, hospital-based design limits the representativeness of COPD patients in the community. Fifth, the voluntary participation system may have resulted in selection bias, selecting patients who were more motivated to quit smoking. Additionally, using self-report data on smoking behaviors and quit intentions may lead to recall bias, and it is possible that participants may be tempted to modify their responses due to social desirability effects. Sixth, the regression model explains a relatively low percentage of variation (14–26%). This could imply important unmeasured factors: psychosocial factors, social support, motivational factors, self-efficacy, etc. We recommend adding these factors in future studies. Seventh, this study did not systematically evaluate potential effect modifiers or interaction effects (e.g. interactions between GOLD stage and prior quit attempts, sex, and additional covariates). Future investigations with sufficient statistical power should examine these interactions to provide deeper insights into the complex relationships among variables. Eight, due to insufficient effect size data and lack of a pilot study for the Smoking Cessation Intention Scale, Cohen’s medium effect size (d=0.5) was assumed for power analysis based on researchers’ experience, which may limit the accuracy of sample size estimation. Ninth, this study’s sample size may be limited in terms of subgroup analyses. Adequate representation across different levels of dependence and demographic characteristics may not have been achieved. Larger sample sizes could enhance statistical power, enabling the detection of smaller effect sizes and facilitating more detailed subgroup comparisons.

## CONCLUSIONS

It was found that the intention to quit smoking was low in patients who were single, those with low symptom scores, patients with early GOLD stage, and those with low knowledge about smoking cessation methods and the health hazards of smoking. Physicians should evaluate the intention of patients with COPD who continue to smoke to quit smoking in a multidimensional way, and consider the associated influencing factors in providing smoking cessation services and referring them to a smoking cessation center. Physicians’ awareness of patients’ intention to quit smoking and the factors that affect it can help them choose the best method based on the individual characteristics of patients. The findings of the study suggest that longitudinal studies focusing on these subgroups are needed to halt the progression of the disease.

## Data Availability

The data supporting this research are available from the authors on reasonable request.
